# CO_2_ Absorption Mechanism by the Deep Eutectic Solvents Formed by Monoethanolamine-Based Protic Ionic Liquid and Ethylene Glycol

**DOI:** 10.3390/ijms23031893

**Published:** 2022-02-08

**Authors:** Jinyu Cheng, Congyi Wu, Weiji Gao, Haoyuan Li, Yanlong Ma, Shiyu Liu, Dezhong Yang

**Affiliations:** 1 School of Earth Sciences and Resources, China University of Geosciences, Beijing 100083, China; 1001191122@cugb.edu.cn (J.C.); 1001191127@cugb.edu.cn (W.G.); 1001191121@cugb.edu.cn (H.L.); 1001191124@cugb.edu.cn (Y.M.); 1001191126@cugb.edu.cn (S.L.); 2 School of Science, China University of Geosciences, Beijing 100083, China; wucongyi@cugb.edu.cn

**Keywords:** capture, CO_2_, deep eutectic solvents, ionic liquids, insights

## Abstract

Deep eutectic solvents (DESs) have been widely used to capture CO_2_ in recent years. Understanding CO_2_ mechanisms by DESs is crucial to the design of efficient DESs for carbon capture. In this work, we studied the CO_2_ absorption mechanism by DESs based on ethylene glycol (EG) and protic ionic liquid ([MEAH][Im]), formed by monoethanolamine (MEA) with imidazole (Im). The interactions between CO_2_ and DESs [MEAH][Im]-EG (1:3) are investigated thoroughly by applying ^1^H and ^13^ C nuclear magnetic resonance (NMR), 2-D NMR, and Fourier-transform infrared (FTIR) techniques. Surprisingly, the results indicate that CO_2_ not only binds to the amine group of MEA but also reacts with the deprotonated EG, yielding carbamate and carbonate species, respectively. The reaction mechanism between CO_2_ and DESs is proposed, which includes two pathways. One pathway is the deprotonation of the [MEAH]^+^ cation by the [Im]^−^ anion, resulting in the formation of neutral molecule MEA, which then reacts with CO_2_ to form a carbamate species. In the other pathway, EG is deprotonated by the [Im]^−^, and then the deprotonated EG, HO-CH_2_-CH_2_-O^−^, binds with CO_2_ to form a carbonate species. The absorption mechanism found by this work is different from those of other DESs formed by protic ionic liquids and EG, and we believe the new insights into the interactions between CO_2_ and DESs will be beneficial to the design and applications of DESs for carbon capture in the future.

## 1. Introduction

Recently, deep eutectic solvents have attracted much attention because of their unique properties, including a negligible vapor pressure, non-flammability, ease of synthesis, and tailorable polarity, which make them a promising alternative to traditional organic solvents [1,2]. Most of DESs are obtained by mixing appropriate hydrogen bond donors (HBDs) and hydrogen bond acceptors (HBAs). The applications of DESs in many fields have been investigated so far, such as organic synthesis, extractions processes, catalytic reactions and electrochemistry [3,4,5,6]. Moreover, DESs have been widely studied as absorbents for CO_2_ capture [7,8,9,10,11]. Among them, functional DESs which could chemically absorb CO_2_ have received much attention because of their high capacities. The chemical absorption of CO_2_ by DESs depends on the chemical reactions between CO_2_ and functional groups of DESs [12,13,14,15,16]. Therefore, understanding the reaction mechanisms between CO_2_ and functional groups of DESs is important to the design of efficient DESs for CO_2_ capture.

Trivedi et al. studied the CO_2_ absorption mechanism by DESs employing ethylenediamine (EDA) as an HBD [17] and the results indicated that a carbamate species was formed between the amine group of EDA and CO_2_. In our previous work, we found that CO_2_ was attached to ethylene glycol (EG) with the formation of a carbonate species when CO_2_ was captured by DESs consisting of azolide-based ILs and EG [18]. Zhang et al. studied the interactions between CO_2_ and ternary DESs consisting of ionic liquid (IL) 1-butyl-3-methylimidazolium chloride ([Bmim][Cl]), imidazole (Im), and super base 1,5-diazabicyclo [4.3.0] non-5-ene (DBN) [19]. The results indicated that CO_2_ was bonded to the C_2_-position of the imidazolium ring of the cation [Bmim]^+^. Zeng et al. investigated the reaction pathway between CO_2_ and DESs composed of protic IL 1,8-diazabicyclo-[0,4,5]undec-7-ene imidazolate ([DBUH][Im]) and EG [20] and it was found that CO_2_ reacted both with the anion [Im]^−^ and EG. Another reaction mechanism between CO_2_ and DESs consisting of ionic liquid 1-ethyl-3-methylimidazolium 2-cyanopyrrolide ([Emim][2-CNpyr]) and EG was reported by Gurkan and co-authors [21], and the results revealed that CO_2_ was attached to the anion [2-CNpyr]-, the imidazolium cation and EG, by forming carbamate, carboxylate and carbonate species, respectively.

In a recent paper, Mukesh et al. reported the CO_2_ capture by DESs based on EG and protic ILs [22]. The protic ILs they used were obtained by mixing polyamines or monoethanolamine (MEA) with imidazole (Im). These DESs showed a high absorption capacity for CO_2_. The authors also investigated the reactions between CO_2_ and the DESs used in their work. On the basis of the NMR and FTIR results, they believed that CO_2_ was bonded to the amine group of polyamines or MEA, and EG or anion [Im]^−^ did not react with CO_2_.

**Scheme 1:**
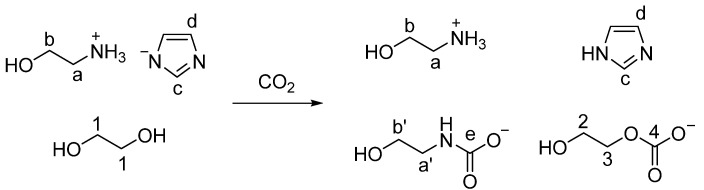
The reaction between CO_2_ and [MEAH][Im]-EG (1:3).

However, in our study, we found that CO_2_ not only binds with the amine group but also reacts with EG in DESs (Scheme 1), suggesting the CO_2_ absorption mechanism reported by Mukesh et al. is inaccurate [22]. The absorption mechanism found by this work is different from those of other DESs formed by protic ionic liquids and EG. For example, CO_2_ reacted both with the anion [Im]^−^ and EG, but did not react with the cation [DBUH]^+^, when CO_2_ was captured by [DBUH][Im]-EG DESs. The CO_2_ absorption mechanism by [DBUH][MLU] (MLU: Methyl urea)-EG presented a similar phenomenon, i.e., CO_2_ reacted with the anion [MLU]^−^ and EG rather than reacting with the cation [DBUH]^+^ [16]. However, the mechanism found by our work indicates that CO_2_ binds to both the amino group on the cation [MEAH]^+^ and EG, but CO_2_ is not bonded to the anion [Im]^−^ in [MEAH][Im]-EG (1:3) DESs. We believe that the findings of our work provide new insights about the interactions between CO_2_ and DESs, which will be useful to design new, efficient DESs for carbon capture in the future.

## 2. Results and Discussion

**Figure 1:**
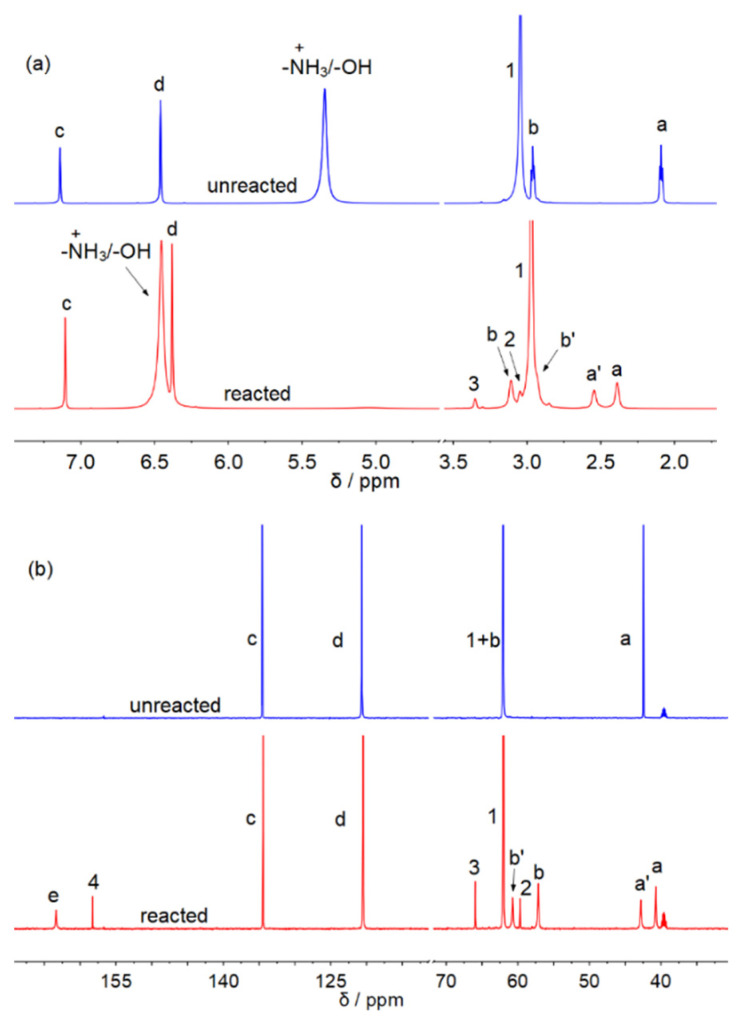
^1^H (**a**) and ^13^C (**b**) NMR spectra of [MEAH][Im]-EG (1:3) before and after CO_2_ absorption.

At first, we prepared the DESs [MEAH][Im]-EG (1:3) following the procedures reported by Mukesh and co-authors [22]. The formation of [MEAH][Im] was confirmed by the ^1^H NMR results (Figure S1). As shown in Figure S1, the -NH_3_^+^ peak can be observed at 5.54 ppm, suggesting the IL [MEAH][Im] was obtained. The CO_2_ capacity by the [MEAH][Im]-EG (1:3) is 8.94 wt% (Figure S2), which is similar to that reported by Mukesh’s paper, and the diagram of the CO_2_ absorption apparatus is shown in Figure S3. The ^1^H (Figure 1a) and ^13^C (Figure 1b) NMR spectra of [MEAH][Im]-EG(1:3) before and after CO_2_ capture were carefully studied. As shown in Figure 1a, the hydrogen peaks of -CH_2_- group (H-a′ and H-b′) of MEA-based carbamate can be found after CO_2_ absorption, [23] suggesting the CO_2_ is bonded to the amine group. However, two other new peaks at 3.05 (H-2) and 3.35 (H-3) ppm can also be found in Figure 1a. As seen in Figure 1b, the carbon signals of -CH_2_- group related to the MEA-based carbamate can be found at 42.7 (C-a′) and 60.6 (C-b′) ppm, and the carbonyl signal of MEA-based carbamate appears at 163.3 (C-e) ppm. However, there are three other new peaks at 60.7 (C-2), 65.9 (C-3), and 158.2 (C-4) ppm.

**Figure 2:**
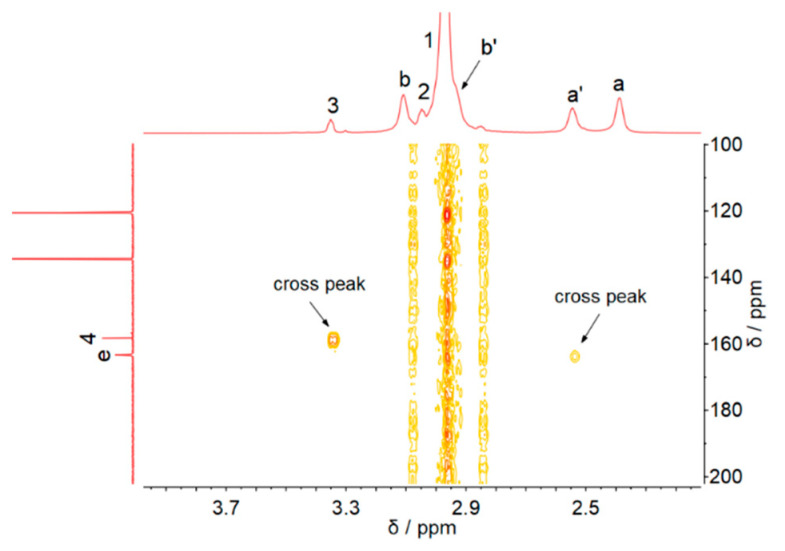
The ^1^H-^13^C HMBC spectra of [MEAH][Im]-EG (1:3) after CO_2_ capture.

In order to assign the new peaks in the ^1^H and ^13^C NMR spectra after CO_2_ capture, the ^1^H-^13^C HMBC NMR spectra of [MEA][Im]-EG (1:3) after CO_2_ capture were investigated (Figure 2). As shown in Figure 2, C-e correlates with the H-a′, suggesting the formation of the MEA-based carbamate. Moreover, the carbonyl carbon C-4 correlates with the H-3, and the correlation between C-4 and the hydrogens on the imidazole ring also could not be seen. The ^1^H-^13^C HMBC suggested that the carbon C-4 should be the carbonyl carbon of the carbonate formed by CO_2_ and EG. The carbons at 60.7 (C-2) and 65.9 (C-3) ppm are the -CH_2_- carbons of HO-CH_2_-CH_2_-OCOO^−^ [20,21]. Similar carbon peaks could also be found in the Mukesh and co-authors’ paper [22]. As shown in Figure S4 of Mukesh and co-authors’ paper, the -CH_2_- carbons of HO-CH_2_-CH_2_-OCOO^−^ could be clearly observed in the ^13^C NMR spectra of [TEPA]_2_[Im]:EG-4 after CO_2_ absorption, which disappeared after CO_2_ desorption. In Figure S10a of their paper, the -CH_2_- carbons of HO-CH_2_-CH_2_-OCOO^−^ could also be found in the ^13^C NMR spectra of TPEA-EG solution after CO_2_ capture, and the carbonyl carbon of EG-based carbonate appeared at 159.67 ppm. In the ^13^C NMR spectra of [TEPA]_2_[Im]:EG-4 after CO_2_ absorption (Figure S10c of their paper), the carbonyl carbon of EG-based carbonate appeared at 159.79 ppm. Therefore, in our opinion, the ^13^C NMR results of [TEPA]_2_[Im]:EG-4 after carbon capture indicated that CO_2_ reacted with EG by forming carbonate species. Interestingly, as seen in Figure 1a, the peaks H-c, H-d, and H-1 shift slightly to upfield, and the H-a peak shift to downfield after carbon capture. These shifts may be due to the change in the hydrogen bonds network in the DESs because of the formations of carbamate and carbonate species after capture.

Although Mukesh and co-authors investigated the HMBC spectra of [TEPA]_2_[Im]:EG-4 after CO_2_ capture (Figure S11 of their paper) and they believed that CO_2_ did not react with EG on the basis of the HMBC results, we think their HMBC results are not accurate because the ^13^C peaks in the HMBC spectra are not consistent with those in the ^13^C NMR spectra of Figure S10c of their paper. In the ^13^C NMR spectra of Figure S10c, the carbamate signals were in the range of 164 to 165 ppm and the strong carbonate signal was at 159.79 ppm. In their HMBC spectra, the carbamate signals can be found near 165 ppm, but the peak near 159 ppm could not be observed, so the HMBC spectra they obtained may be incorrect.

**Figure 3:**
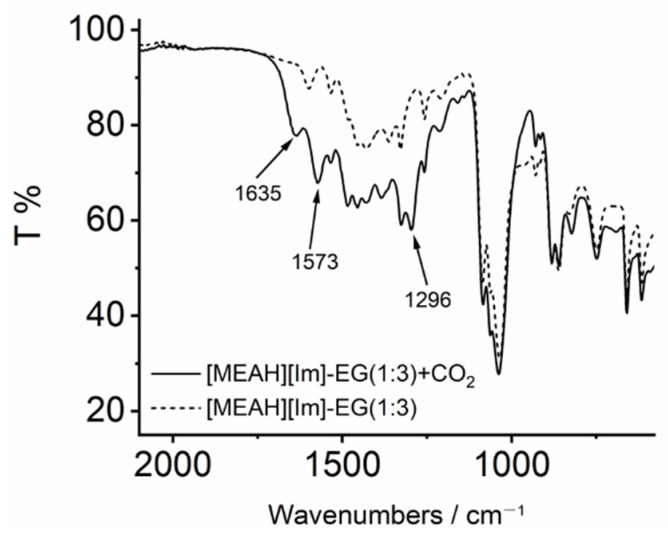
The FTIR spectra of [MEAH][Im]-EG (1:3) before and after CO_2_ capture.

Our findings were further confirmed by the FTIR spectra. The FTIR spectra of [MEAH][Im]-EG (1:3) before and after CO_2_ capture were shown in Figure 3. The peak at 1635 cm^−1^ is associated with the combined C=O stretching band of carbonate and carbamate species, and the peak at 1573 cm^−1^ corresponds to COO^−^ asymmetric stretching of carbamate -NHCOO^−^. The new peak at 1296 cm^−1^ is ascribed to the stretching band of RO-COO^−^ [21]. The FTIR results suggested again that both carbonate and carbamate species are formed after CO_2_ capture.

**Scheme 2:**
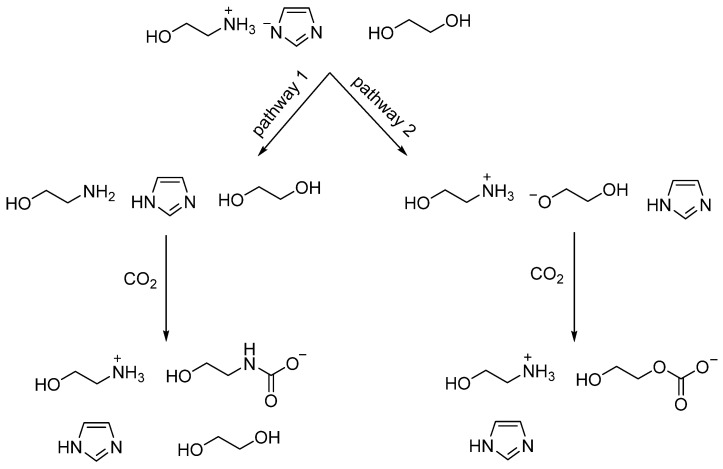
The proposed reaction mechanism between CO_2_ and [MEAH][Im]-EG (1:3).

Based on the above discussion, the possible absorption mechanism by [MEAH][Im]-EG (1:3) is presented in Scheme 2. Pathway 1 is the deprotonation of the [MEAH]^+^ cation by [Im]^−^ anion, resulting in the formation of the neutral molecule MEA, which then reacts with CO_2_ to form a carbamate species. Pathway 2 is that EG is deprotonated by [Im]^−^, forming the HO-CH_2_-CH_2_-O^−^ anion, which then binds with CO_2_ to form a carbonate species. The absorption mechanism by [MEAH][Im]-EG (1:3) is different from that of [DBUH][Im]-EG, which may be due to that DBU is a much stronger base than MEA. In other words, [DBUH]^+^ is a much weaker acid than [MEAH]^+^, rendering the deprotonation of [DBUH]^+^ by [Im]^−^ extremely difficult, so CO_2_ is not found to be bonded to DBU in the [DBUH][Im]-EG DESs after CO_2_ capture [20]. In our previous work, the steric hindrance of functional groups is also found to be a factor that affects the CO_2_ absorption mechanism. [24,25]. Therefore, we suggest that the factors, which may include the basicity, acidity, and the steric hindrance of functional groups of DESs, should be considered to modulate the CO_2_ absorption mechanism by DESs.

## 3. Materials and Methods

### 3.1. Materials and Characterizations

Imidazole (Im, 98%), ethylene glycol (EG, 99.5%), and monoethanolamine (MEA, 99%) were obtained from J&K Scientific Ltd. (Beijing, China). CO_2_ (≥99.99%) was supplied by Beijing ZG Special Gases Sci. and Tech. Co. Ltd. The ^1^H NMR (600 MHz) and ^13^C NMR (151 MHz) spectra were recorded on a Bruker spectrometer using DMSO-d_6_ as an external solvent. FTIR spectra were recorded on a PerkinElmer Frontier spectrometer with an attenuated total reflection (ATR) accessory. The water content of the DESs was determined using Karl Fisher titration by V20 Volumetric KF titrator (Mettler Toledo, Zaventem, Belgium).

### 3.2. Synthesis of [MEAH][Im] and DESs

The procedure to prepare [MEAH][Im] followed the methods reported by Mukesh et al. MEA and Im were mixed in a flask at a 1:1 molar ratio, and then the mixtures were stirred at 80 °C for 2 h. The obtained [MEAH][Im] was stored in a glass vial.

The DESs [MEAH][Im]-EG (1:3) was obtained by mixing [MEAH][Im] and EG in a glass vial at a 1:3 molar ratio at room temperature. The water content of [MEAH][Im]-EG (1:3) is 0.23 wt%.

### 3.3. CO_2_ Absorption

[MEAH][Im]-EG (1:3) (2.0 g) was added into a glass tube with a diameter of 10 mm. The tube was equipped with a rubber lid, which contained two needles. The tube was partially immersed in a water bath (25 ± 0.3 °C), and CO_2_ was bubbled into the solvent through one needle at a flow rate of ~50 mL/min, and the other needle was for a CO_2_ outlet. The weight difference of the tube before and after CO_2_ absorption was determined by an electronic balance (±0.1 mg).

## 4. Conclusions

The reaction mechanism between CO_2_ and DES [MEA][Im]-EG (1:3) is studied carefully by using NMR and FTIR spectroscopy. The CO_2_ capacity of [MEA][Im]-EG (1:3) is 8.94 wt% at 25 °C and 1.0 atm. The results indicate that CO_2_ not only binds with the amino group of MEA but also reacts with the deprotonated EG in the solvent. There are two possible reaction pathways that form carbamate and carbonate species. One is the deprotonation of the [MEAH]^+^ cation by [Im]^−^ anion, forming the neutral molecule MEA, which then reacts with CO_2_ to form a carbamate species. Another one is the reaction between EG and [Im]^−^, forming the HO-CH_2_-CH_2_-O^−^ anion, which then binds with CO_2_ to form a carbonate species. We believe the new insights about the interactions between CO_2_ and DESs will bring benefits to the design and applications of DESs for carbon capture in the future.

## Data Availability

Not applicable.

## Supplementary Materials

The following supporting information can be downloaded at: https://www.mdpi.com/article/10.3390/ijms23031893/s1.
