# Supplementary material for: CO2 Absorption Mechanism by the Deep Eutectic Solvents Formed by Monoethanolamine-Based Protic Ionic Liquid and Ethylene Glycol
Source: Int J Mol Sci. 2022 Feb 8;23(3):1893. doi: 10.3390/ijms23031893 (PMC8836646; doi:10.3390/ijms23031893)
Supplement: Supplementary file 1 [file ijms-23-01893-s001.zip › ijms-1567929-supplementary.pdf]

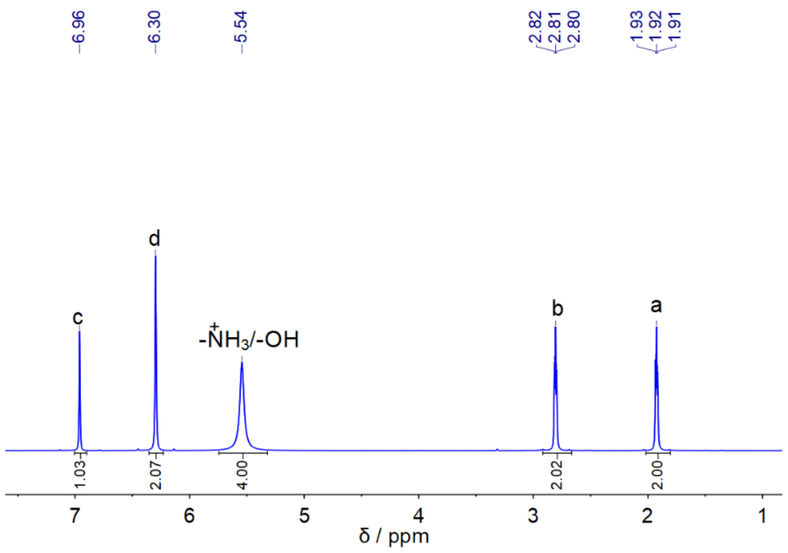

Figure S1. The ¹H NMR spectra of [MEAH][Im].

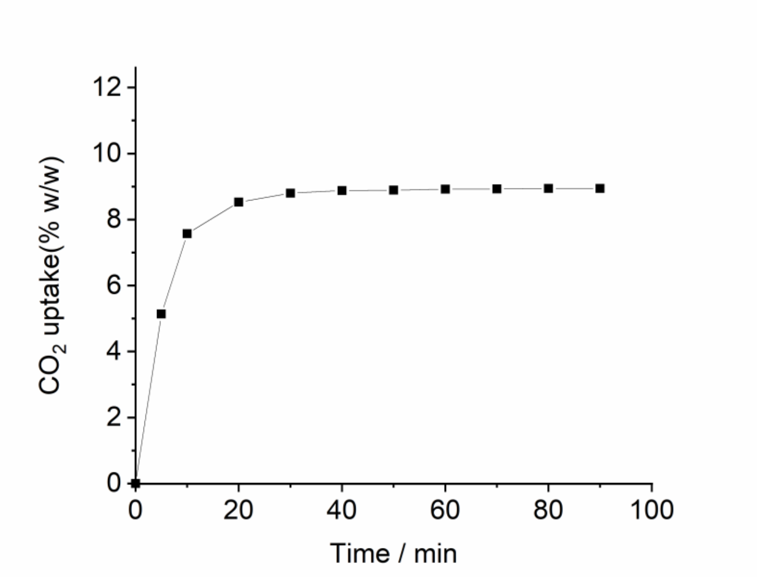

Figure S2. CO_2 absorption by [MEAH][Im]-EG (1:3) at 25 ℃ and 1.0 atm.

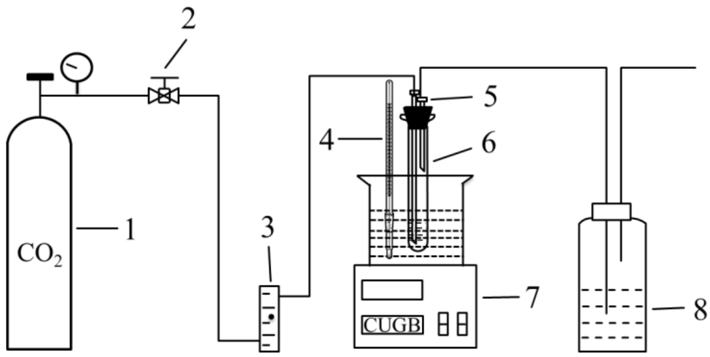

Figure S3. Schematic diagram of CO_2 absorption apparatus. 1, gas cylinder; 2, needle valve; 3, gas flowmeter; 4, thermometer; 5, needle; 6, glass tube; 7, digital thermostat water bath; 8, NaOH aqueous solution.
